# Pulmonary inflammation and cytokine dynamics of bronchoalveolar lavage fluid from a mouse model of bronchial asthma during A(H1N1)pdm09 influenza infection

**DOI:** 10.1038/s41598-017-08030-w

**Published:** 2017-08-22

**Authors:** Yousuke Fujimoto, Shunji Hasegawa, Takeshi Matsushige, Hiroyuki Wakiguchi, Tamaki Nakamura, Hideki Hasegawa, Noriko Nakajima, Akira Ainai, Atsunori Oga, Hiroshi Itoh, Komei Shirabe, Shoichi Toda, Ryo Atsuta, Tsuneo Morishima, Shouichi Ohga

**Affiliations:** 1 Department of Pediatrics, Yamaguchi University Graduate School of Medicine, 1-1-1 Minamikogushi, Ube Yamaguchi, 755-8505 Japan; 2 Department of Pathology, National Institute of Infectious Diseases, 1-23-1 Toyama, Shinjuku-ku Tokyo, 162-8640 Japan; 3 Department of Pathology, Yamaguchi University Graduate School of Medicine, 1-1-1 Minamikogushi, Ube Yamaguchi, 755-8505 Japan; 4 Yamaguchi Prefectural Institute of Public Health and Environment, 2-5-67 Aoi, Yamaguchi, 753-0821 Japan; 5 Depertment of Respiratory Medicine, Juntendo Tokyo Koto Geriatric Medical Center, 3-3-20 Shinsuna, Koto-ku Tokyo, 136-0075 Japan; 6 Department of Pediatrics, Okayama University Graduate School of Medicine, Dentistry and Pharmaceutical Sciences, 2-5-1 Shikata-cho, Kita-ku Okayama, 700-8558 Japan

## Abstract

Asthmatic patients present more rapid progression of respiratory distress after A(H1N1)pdm09 influenza infection than after seasonal infection. Here, we sought to clarify the pathophysiology of early deterioration in asthmatic patients after A(H1N1)pdm09 infection. Cytokine levels and virus titres in bronchoalveolar lavage fluid from mice with and without asthma after A(H1N1)pdm09 or seasonal H1N1 infection were examined. In asthma/A(H1N1)pdm09 mice, IL-6 and TNF-α levels peaked at 3 days post-infection and were higher than those in all other groups. IFN-γ levels in asthma/A(H1N1)pdm09 mice at 3 days post-infection were higher than in all other mice at any time point, whereas at 7 days post-infection, the levels were lowest in asthma/A(H1N1)pdm09 mice. Virus titres in asthma/A(H1N1)pdm09 mice were highest at 3 days post-infection, and decreased by 7 days post-infection, although the levels at this time point were still higher than that in any other group. Histopathological examination showed more inflammatory cell infiltration and lung tissue destruction in the asthma/A(H1N1)pdm09 group than in any other group. The distinct cytokine profiles in A(H1N1)pdm09-infected asthmatic mice indicated excessive inflammation and virus replication within a few days after infection. Thus, bronchial asthma could be a more exacerbating factor for pandemic influenza infection than for seasonal influenza infection.

## Introduction

During the global 2009 H1N1 pandemic [A(H1N1)pdm09], there were significantly higher infection rates in children, and approximately 80% of deaths due to A(H1N1)pdm09 infection occurred in individuals aged < 65 years^1^. Although the clinical manifestations of A(H1N1)pdm09 and seasonal H1N1 infections were similar^2^, many severe and fatal cases of A(H1N1)pdm09 occurred not only in patients with underlying diseases but also in healthy children and young adults^3, 4^. Some reports have shown that bronchial asthma was one of the most common underlying conditions in patients hospitalized with A(H1N1)pdm09 infection^5, 6^. Additionally, it has been reported that children with asthma showed increased susceptibility to A(H1N1)pdm09 viral infection^7^, and the incidence of A(H1N1)pdm09 viral infection was significantly higher in children with asthma than in children without asthma^7^.

Paediatric patients with A(H1N1)pdm09 infection showed milder symptoms than those with seasonal H1N1 infection. However, severe respiratory issues, including pneumonia and acute respiratory distress syndrome (ARDS), have been reported in children and young adults with A(H1N1)pdm09 infection^5, 8–10^. Bronchial asthma increases the risk of hospital and intensive care admission in infants and children^3–6, 10–14^. We previously reported that A(H1N1)pdm09 infection, but not seasonal H1N1 infection, induces severe pulmonary inflammation in a mouse model of asthma 7 days after infection^14, 15^. However, we observed that the duration of the latent period for A(H1N1)pdm09 infection is shorter than 7 days, and patients present earlier progression of pulmonary disease and systemic conditions after infection. Since exacerbation was frequently observed in asthmatic patients after viral infection, we suspect that a cytokine storm, including inflammatory cytokines (interleukin [IL]-6 and tumour necrosis factor [TNF]-α), anti-inflammatory cytokines (IL-10), anti-viral cytokines (interferon [IFN]-γ), and helper T (Th) 2 cytokines (IL-4 and IL-13), may occur in the lung following A(H1N1)pdm09 infection. However, little is known about the early host response against A(H1N1)pdm09 infection in patients with underlying bronchial asthma.

In the present study, we investigated the sequential changes in intra-tracheal cytokine production, viral loads, and pulmonary inflammation in a mouse model of bronchial asthma during the first 7 days after A(H1N1)pdm09 or seasonal H1N1 influenza infection.

## Results

### Inflammatory cytokine concentrations in bronchoalveolar lavage (BAL) fluid

**Figure 1:**
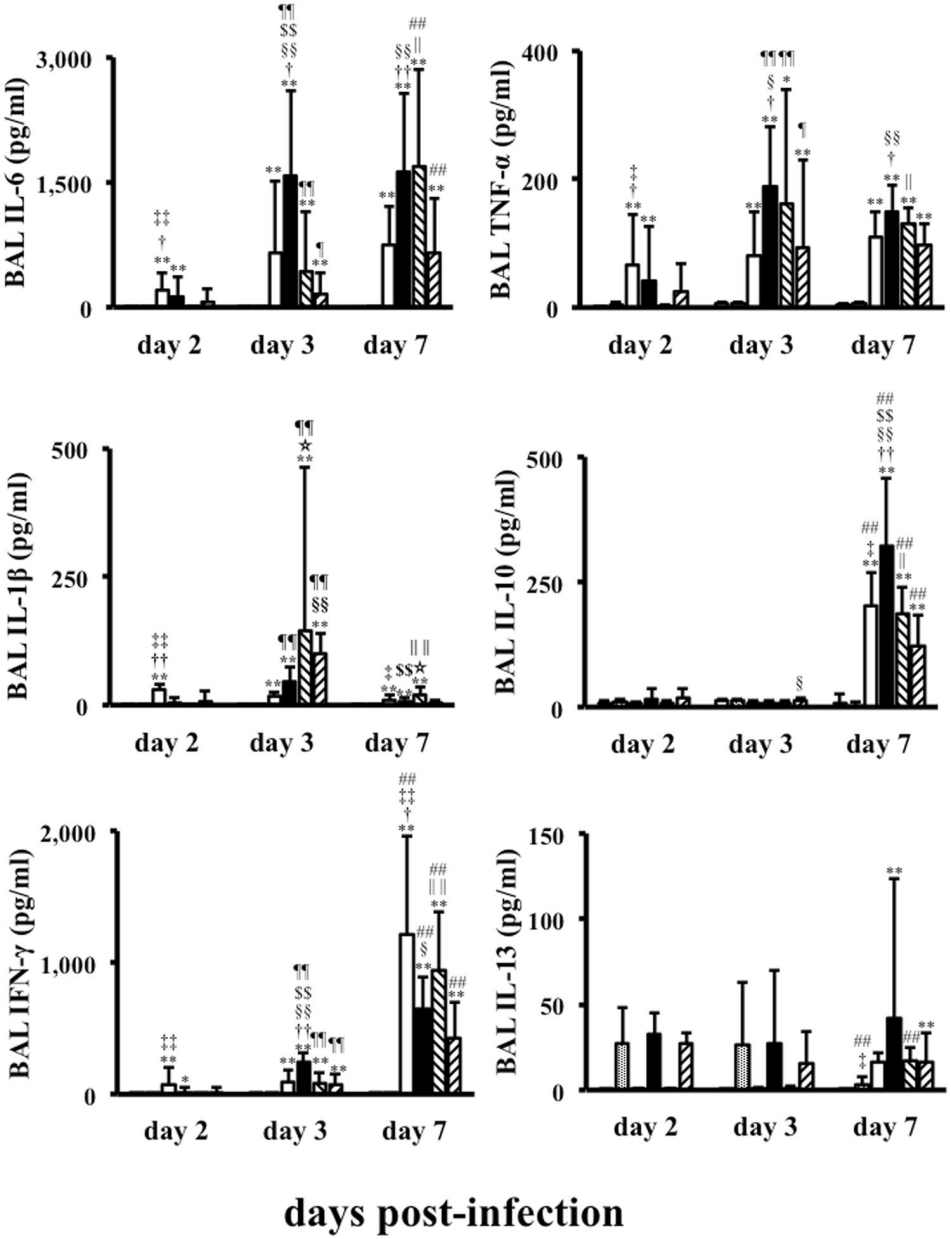
Cytokine levels in bronchoalveolar lavage (BAL) fluid after influenza infection. The levels of interleukin (IL)-6, tumour necrosis factor (TNF)-α, IL-1β, interferon (IFN)-γ, IL-13, and IL-10 in BAL fluids from control and asthmatic mice infected with A(H1N1)pdm09 or seasonal H1N1 (A/Puerto Rico) or mock infected at 2, 3, and 7 days post infection. Data shown are the mean ± standard deviation (SD) of three independent experiments. : control/A(H1N1)pdm09, : asthma/A(H1N1)pdm09, : control/seasonal, : asthma/seasonal, : control/mock, : asthma/mock.  Control/A(H1N1)pdm09 vs. asthma/A(H1N1)pdm09: ^†^ *p* < 0.05, ^††^ *p* < 0.01; control/seasonal vs. asthma/seasonal: || *p* < 0.05, || || *p* < 0.01; control/A(H1N1)pdm09 vs. control/seasonal: *p* < 0.05; asthma/A(H1N1)pdm09 vs. asthma/seasonal: ^§^ *p* < 0.05, ^§§^ *p* < 0.01; asthma/A(H1N1)pdm09 vs. control/seasonal: ^$$^ *p* < 0.01; and control/A(H1N1)pdm09 vs. asthma/seasonal: ^‡^ *p* < 0.05, ^‡‡^ *p* < 0.01; mock/control or mock/asthma vs. each group: *p < 0.05, **p < 0.01; day 2 vs. day 3, ^¶¶^ *p* < 0.01; day 3 vs. day 7: ^##^ *p* < 0.01.

IL-6, TNF-α, and IL-1β concentrations in BAL fluid obtained 2, 3, and 7 days post-infection are shown in Fig. 1. Although the mean IL-6 levels in asthmatic mice challenged with A(H1N1)pdm09 were low (118.9 pg/mL) at 2 days post-infection, the levels were markedly increased (to 1578.2 pg/mL) at 3 days post-infection, and the levels in all groups remained high at 7 days post-infection. In contrast, IL-6 levels in A(H1N1)pdm09-challenged control mice were 198.6 pg/mL at 2 days post-infection, peaked at 463.5 pg/mL by 3 days post-infection, and remained at similar levels at 7 days post-infection.

After challenge with influenza A/Puerto Rico, IL-6 levels in asthmatic mice slowly increased to 161.7 pg/mL by 3 days post-infection, and then reached 654.7 pg/mL at 7 days post-infection. In contrast, IL-6 levels in control mice increased to 433.2 pg/mL at 3 days post-infection, similar to the levels after A(H1N1)pdm09 infection. IL-6 levels in control mice at 3 days post-infection exceeded those of asthmatic mice at this time point (*p *= 0.015), and peaked to 1696.8 pg/mL at 7 days post-infection.

The TNF-α levels in asthmatic/A(H1N1)pdm09 mice increased to 188.5 pg/mL at 3 days post-infection, which was the highest for all groups, and levels remained high at 7 days post-infection (*p *= 0.33). In contrast, TNF-α levels in the control/A(H1N1)pdm09 group increased to 63.7 pg/mL at 3 days post-infection, and peaked to 110.7 pg/mL by 7 days post-infection (*p* = 0.12).

After seasonal virus infection, TNF-α levels in asthmatic mice increased to only 92.2 pg/mL at 3 days post-infection, which was similar to the levels in control/A(H1N1)pdm09 mice (*p* = 0.06), and these levels were maintained until 7 days post-infection. In contrast, the levels in control mice increased to 161.4 pg/mL by 3 days post-seasonal virus infection, which were similar to those in asthma/A(H1N1)pdm09 mice (*p* = 1.00), and these levels were maintained until 7 days post-infection. Elevations in IL-6 and TNF-α levels in BAL fluid were not observed in the two mock-infected groups.

The BAL IL-1β levels in control (145.6 pg/mL) and asthmatic mice (100.7 pg/mL) after seasonal infection were significantly higher than the A(H1N1)pdm09-infected groups (control/A(H1N1)pdm09: 16.5 pg/mL, asthmatic/A(H1N1)pdm09: 45.0 pg/mL), respectively.

The early increasing pattern of IL-6 and TNF-α levels (but not IL-1β) in asthmatic mice after A(H1N1)pdm09 infection (but not control mice), was in contrast to the dynamics observed in A/Puerto Rico-infected mice.

### Other cytokines in BAL fluid

IFN-γ levels in asthmatic/A(H1N1)pdm09 mice significantly increased to 240.4 pg/mL by 3 days post-infection, which was the highest among all mice at this time point (vs. control/A(H1N1)pdm09, *p* = 0.007; vs. asthma/seasonal, *p* = 0.002; vs. control/seasonal, *p* = 0.001), and then levels increased to 651.5 pg/mL at 7 days post-infection (*p* = 0.001). IFN-γ levels in control mice at 3 days after A(H1N1)pdm09 infection increased to 70.8 pg/mL, which was significantly lower than the levels in asthmatic/A(H1N1)pdm09 mice at 3 days post-infection (*p* = 0.007). However, IFN-γ levels in control/A(H1N1)pdm09 mice peaked at 1209.4 pg/mL at 7 days post-infection, which were the highest of all the groups.

IL-10 levels were undetectable in all mice until 3 days post-infection. At 7 days post-infection, the levels in asthmatic/A(H1N1)pdm09 mice increased to 322.1 pg/mL, which was the highest of all the groups (vs. control/A(H1N1)pdm09, *p* = 0.007; vs. asthma/seasonal, *p* = 0.0001; vs. control/seasonal, *p* = 0.005). IL-10 levels in control/A(H1N1)pdm09 mice increased to 202.4 pg/mL at 7 days post-infection. These levels were similar to those in control/seasonal mice (185.3 pg/mL, *p* = 0.44) but higher than those in asthma/seasonal mice (122.9 pg/mL, *p* = 0.03).

After seasonal virus infection, the IFN-γ levels in asthmatic mice increased to 72.8 pg/mL at 3 days post-infection, which were similar to the levels in A/Puerto Rico-challenged control and asthmatic mice at 3 days post-infection, but were lower than those in asthmatic/A(H1N1)pdm09 mice at 3 days post-infection (*p* = 0.002). The IFN-γ levels in asthmatic/seasonal mice then increased to 420.6 pg/mL by 7 days post-infection, which were lower than that in any other group at this time point (vs. asthmatic/A(H1N1)pdm09, *p* = 0.03; vs. control/A(H1N1)pdm09, *p* = 0.002; and vs. control/seasonal, *p* = 0.002). IFN-γ levels in control/seasonal mice only increased to 80.7 pg/mL at 3 days post-infection, but then increased to 945.0 pg/mL by 7 days post-infection (*p* = 0.0001). Neither IL-10 levels nor IFN-γ levels were elevated in non-infected groups.

BAL IL-13 levels in all asthmatic groups were higher than those in all non-asthmatic control groups, but the differences were not statistically significant. Additionally, the levels in the control/A(H1N1)pdm09 and asthma/A(H1N1)pdm09 groups increased from 3 to 7 days post-infection.

No significant differences in the levels of IL-2, IL-4, or IL-17A, were observed among the groups, and the levels were all below the detection limits.

### Cell infiltration in BAL fluid

**Figure 2:**
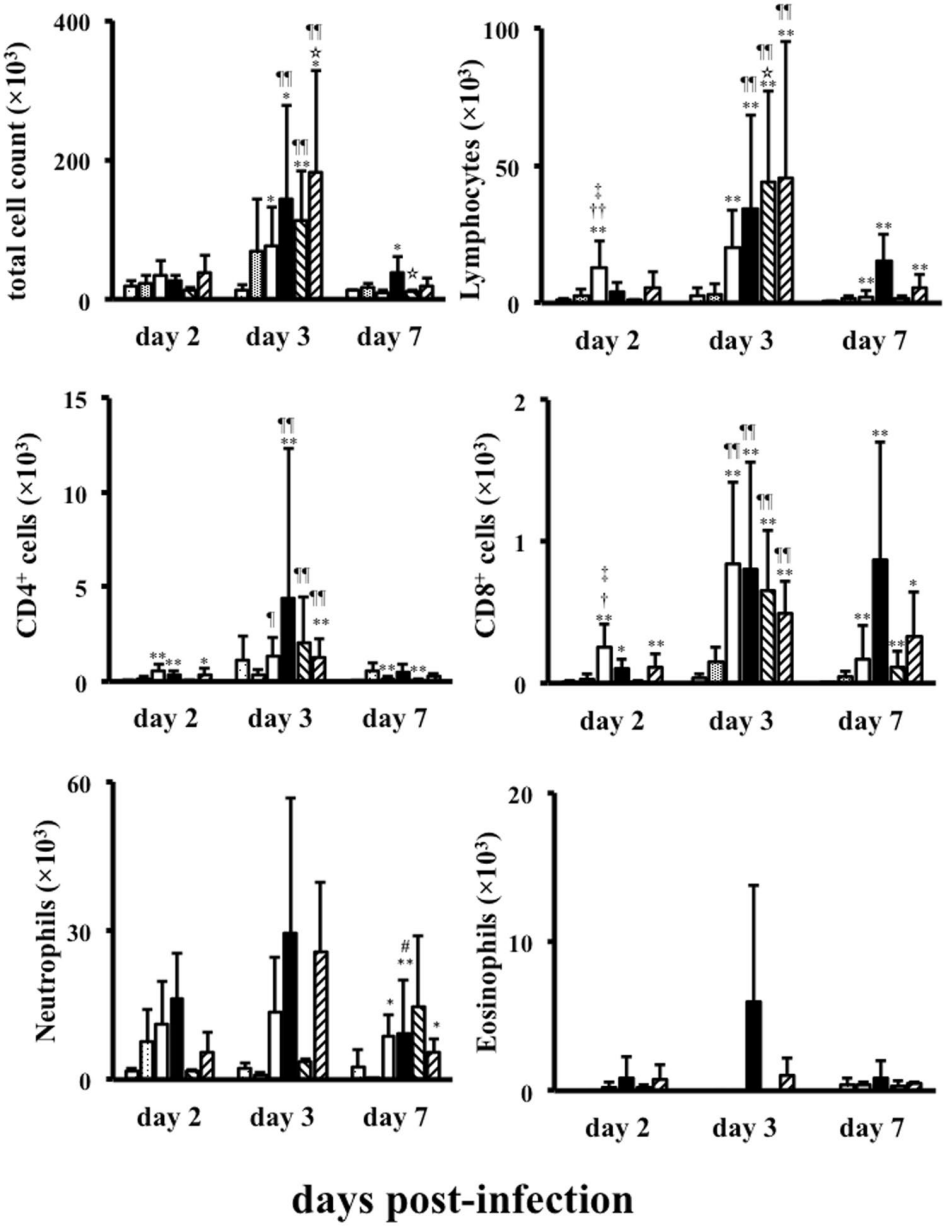
The number of infiltrated cells in BAL fluid after influenza infection. Cell infiltration in BAL fluid from control and asthmatic mice infected with vehicle (mock), A(H1N1)pdm09, or seasonal H1N1 (A/Puerto Rico) at 2, 3, and 7 days post-infection as determined by flow cytometry and cytospin samples. Data are the mean ± standard deviation (SD) of three independent experiments. : control/A(H1N1)pdm09, : asthma/A(H1N1)pdm09, : control/seasonal, : asthma/seasonal, : control/mock, : asthma/mock.  Control/A(H1N1)pdm09 vs. asthma/A(H1N1)pdm09: ^†^ *p* < 0.05, ^††^ *p* < 0.01; control/seasonal vs. asthma/seasonal: **||** *p* < 0.05, **|| ||** *p* < 0.01; control/A(H1N1)pdm09 vs. control/seasonal: *p < 0.05; asthma/A(H1N1)pdm09 vs. asthma/seasonal: ^§^ *p* < 0.05, ^§§^ *p* < 0.01; asthma/A(H1N1)pdm09 vs. control/seasonal: ^$$^ *p* < 0.01; and control/A(H1N1)pdm09 vs. asthma/seasonal: ^‡^ *p* < 0.05, ^‡‡^ *p* < 0.01; mock group vs. each group: *p < 0.05, **p < 0.01; day 2 vs. day 3, ^¶¶^ *p* < 0.01; day 3 vs. day 7: ^##^ *p* < 0.01.

The differences in the numbers of inflammatory cells in BAL fluid from mice in each of the groups are shown in Fig. 2. The numbers of total cells, lymphocytes, CD4^+^ cells, and CD8^+^ cells in all infected groups were increased at 3 days post-infection, and then decreased at 7 days post-infection. The numbers of CD8^+^ cells in the asthma/A(H1N1)pdm09 and control/seasonal groups were maintained until 7 days post-infection. The number of lymphocytes in control/seasonal group were higher than that in control/A(H1N1)pdm09 at 3 days post-infection, however, there were no significant differences between control/seasonal and asthma/A(H1N1)pdm09, control/A(H1N1)pdm09 and asthma/A(H1N1)pdm09 groups, respectively. Additionally, the numbers of CD4^+^ cells, neutrophils and eosinophils on the 3 days and CD8^+^ cells on the 7 days post-infection were higher in the asthmatic/A(H1N1)pdm09 group than other groups respectively, but not statistically. In contrast, the numbers in the control/mock and asthma/mock groups were lower than those in the infected groups.

### Virus titres in BAL fluid

**Figure 3:**
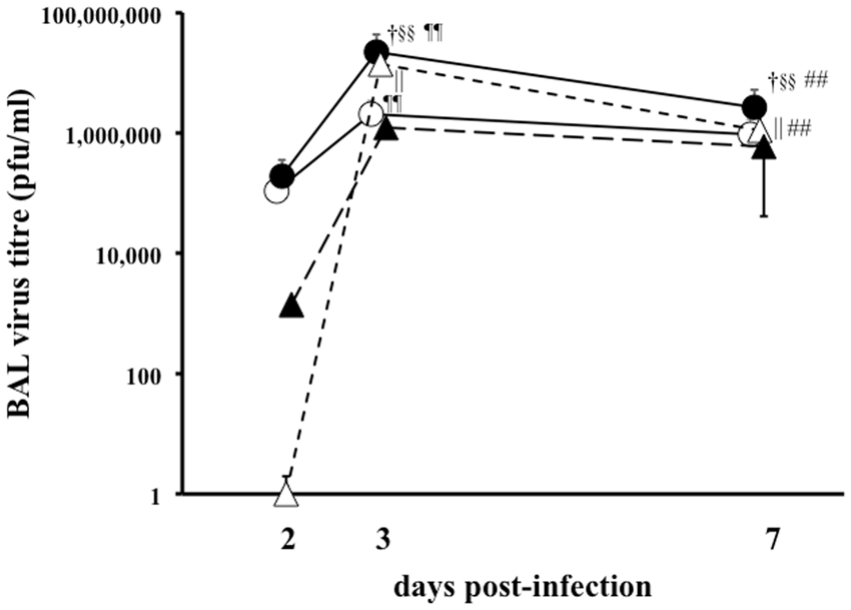
Virus titres in BAL fluid after influenza infection. The virus titres in BAL fluid from control and asthmatic mice infected with A(H1N1)pdm09 or seasonal H1N1 (A/Puerto Rico) at 2, 3, and 7 days post-infection, as determined by the plaque assay. ○: control/A(H1N1)pdm09, ●: asthma/A(H1N1)pdm09, △: control/seasonal, ▲: asthma/seasonal. Data are the mean ± SD of three independent experiments. Control/A(H1N1)pdm09 vs. asthma/A(H1N1)pdm09: ^†^ *p* < 0.05, ^††^ *p* < 0.01; control/seasonal vs. asthma/seasonal: ||*p* < 0.05, || ||*p* < 0.01; asthma/A(H1N1)pdm09 vs. asthma/seasonal: ^§^ *p* < 0.05, ^§§^ *p* < 0.01; day 2 vs. day 3, ^¶¶^ *p* < 0.01; day 3 vs. day 7: ^##^ *p* < 0.01.

The virus titres in BAL fluid at 2, 3, and 7 days post-infection are shown in Fig. 3. The mean titres at 2 days post-infection in the A(H1N1)pdm09-infected groups (asthma/A(H1N1)pdm09: 1.9 × 10^5^ pfu/mL and control/A(H1N1)pdm09: 1.1 × 10^5^ pfu/mL) were higher than those in the seasonal-infected groups (asthma/seasonal: 1.4 × 10^4^ pfu/mL, control/seasonal: not detected); however, these differences were not statistically significant. The mean titre in the asthmatic/A(H1N1)pdm09 group at 3 days post-infection (2.2 × 10^7^ pfu/mL) was the highest of all groups, and the differences were significant (vs. control/A(H1N1)pdm09, *p* = 0.001; vs. asthma/seasonal, *p* = 0.001), with the exception of the control/seasonal group. The virus titre in the asthmatic/A(H1N1)pdm09 group at 7 days post-infection (2.6 × 10^6^ pfu/mL) was higher than those in any other groups at this time point (vs. control/A(H1N1)pdm09, *p *= 0.03; vs. asthma/seasonal, *p* = 0.001; vs. control/seasonal, *p* = 0.03). After A(H1N1)pdm09 infection, the titre in asthmatic mice at 3 days post-infection was higher than the titre at 7 days post-infection (*p* = 0.0002). The virus titres in control mice were also higher at 3 days post-infection than at 7 days post-infection (*p* = 0.006) after challenge with seasonal H1N1.

### Histopathological findings in the lungs

**Figure 4:**
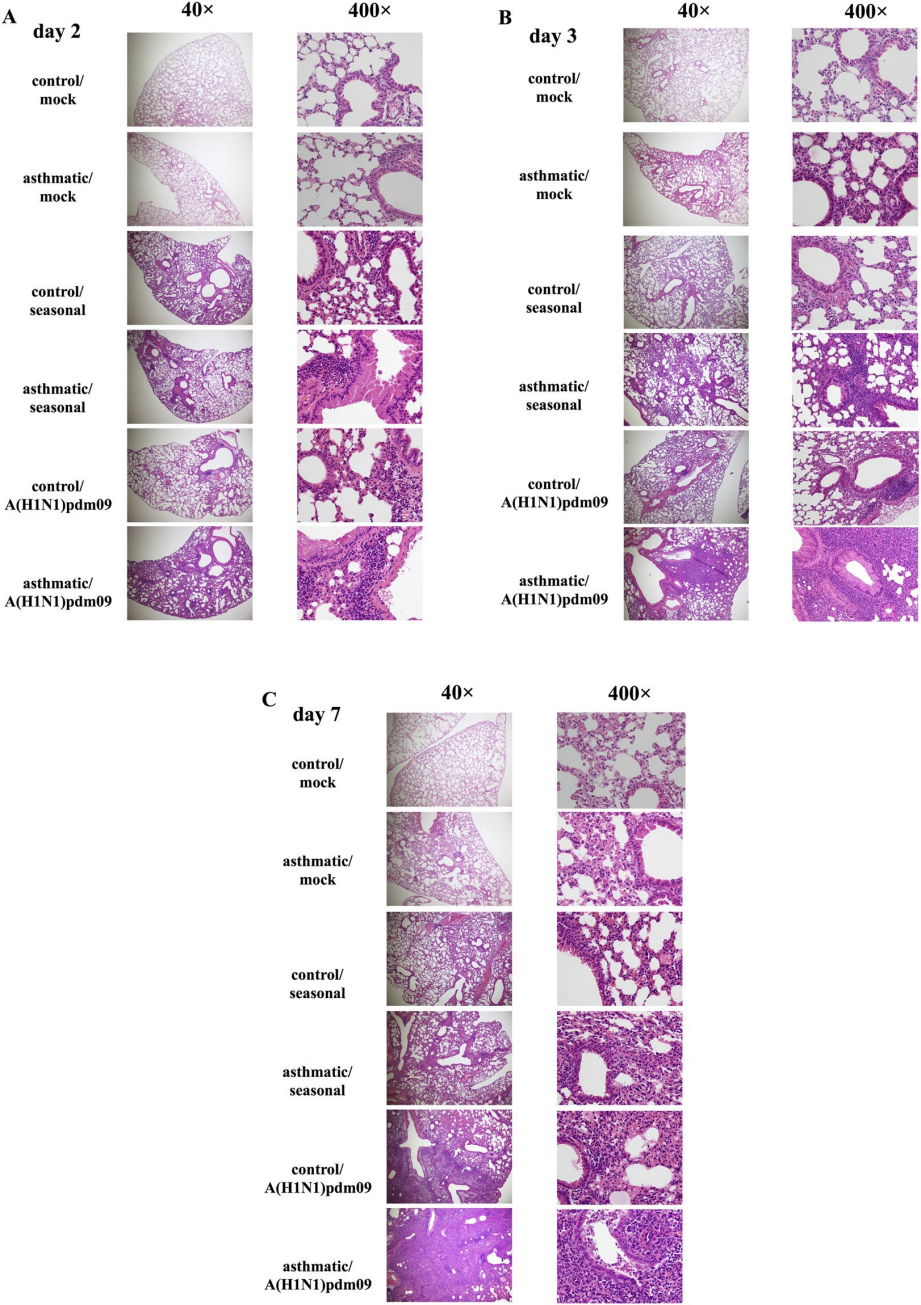
Histopathological findings after influenza infection. Photomicrographs of hematoxylin and eosin (H&E)-stained lung tissue at 2 (**A**), 3 (**B**), and 7 (**C**) days post-infection with mock, seasonal H1N1 (A/Puerto Rico) or A(H1N1)pdm09 influenza virus. Similar results were obtained in six independent mice from each group. Representative findings are shown.

Figure 4 shows the H&E staining of lung tissues from mice at 2 (**A**), 3 (**B**) and 7 (**C**) days post-infection. The degrees of inflammatory cell infiltration and abscess formation in the asthma/A(H1N1)pdm09 group were more remarkable than in the control/A(H1N1)pdm09, asthma/seasonal, and control/seasonal groups on 2, 3 and 7 days post-infection. In addition, on 7 days post-infection, they were most severe in asthma/A(H1N1)pdm09 mice, compared with other mice.

### Immunohistochemistry for the distribution of A(H1N1)pdm09

**Figure 5:**
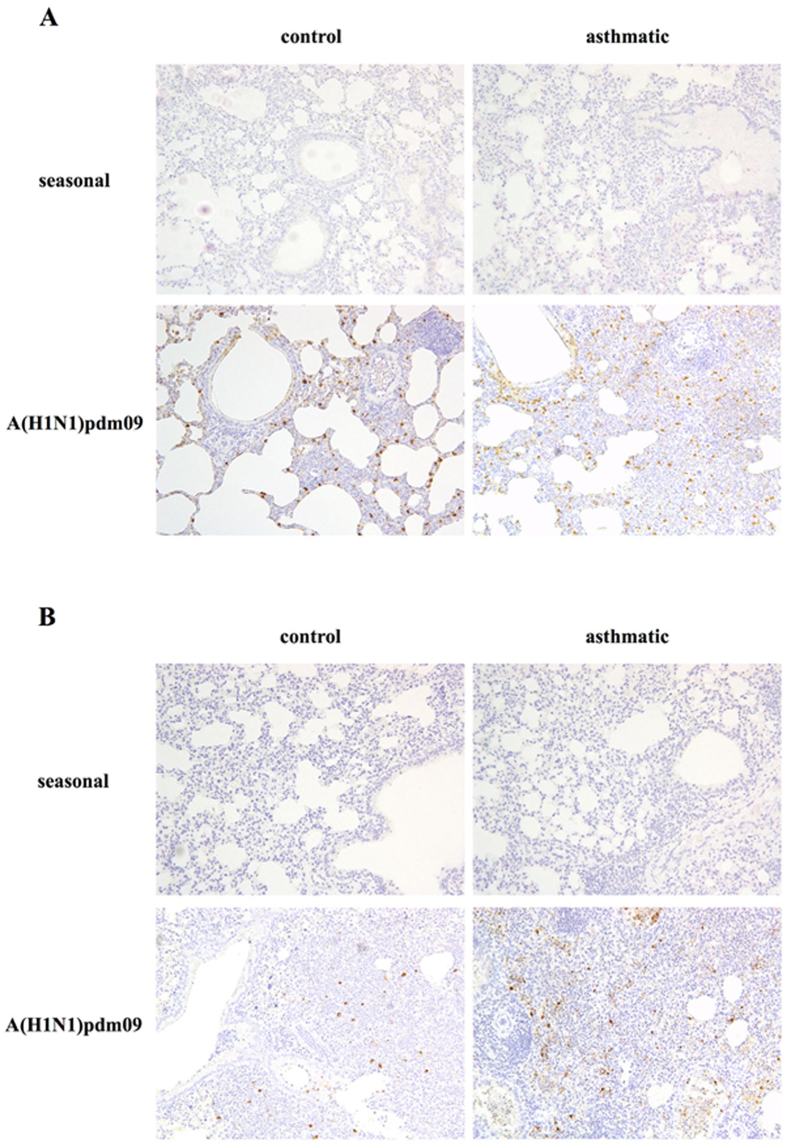
Immunohistochemical findings after influenza infection. Photomicrographs of immunohistochemistry assays of lung tissue in early phase (**A**), control/A(H1N1)pdm09: day 2, others: day 3), and day 7 (**B**) post-infection with seasonal or A(H1N1)pdm09 influenza virus (×200). Similar results were obtained in two independent mice from each group. Representative findings are shown.

The distribution of type A influenza nucleoprotein antigen (InfA-NP) in the lungs of mice after A(H1N1)pdm09 or seasonal infection were observed by immunohistochemistry (Fig. 5). InfA-NP was detected in the epithelial cells and suspected macrophages in the lungs of asthmatic/A(H1N1)pdm09 (**A**, day 3) and control/A(H1N1)pdm09 group mice (**A**, day 2) since the early phase to day 7 (**B**) after infection, but not in mice from seasonal infected groups. Infiltration of various inflammatory cells was noted, mainly in the alveolar walls in all four infected groups and more around the bronchioles in the A(H1N1)pdm09-infected groups, than in the seasonal H1N1-infected groups. Only the asthmatic/A(H1N1)pdm09 group showed abscess formation with severe inflammation.

## Discussion

The notable findings in the present study were the early peak in both IL-6 and TNF-α levels, the high inflammatory cell infiltration in BAL fluids, and the severe pulmonary inflammation at 3 days post-infection in asthmatic/A(H1N1)pdm09 mice. The pulmonary cytokine storm at 3 days post-infection in asthma/A(H1N1)pdm09 mice may mirror the rapid exacerbation observed in asthmatic patients^13^. In contrast, the delayed peak in IL-10 levels and insufficient surge of IFN-γ levels in A(H1N1)pdm09 mice at 7 days post-infection could lead to ineffective exclusion of the viruses. The early potent inflammation associated with high viral loads in the lungs of asthmatic/A(H1N1)pdm09 mice may corroborate the rapid progression of asthmatic patients during outbreaks of pandemic virus infection. Because the dynamics of IL-1β was different from those of IL-6 and TNF-α levels, IL-1β may play other roles after influenza virus infection. In addition, the IL-13 levels were increased in only the asthma/A(H1N1)pdm09 group after the infection. IL-13 may be involved in pathophysiology of A(H1N1)pdm09 infection in asthmatic children. Although IL-2, IL-4, and IL-17A may be involved in the pathogenesis of bronchial asthma and influenza infection, they were undetectable in the BAL fluid from mice in this study. This may be explained by the cytokines’ short half-lives and/or limited roles in this microenvironment.

In asthma/A(H1N1)pdm09 group, the number of CD4^+^ cells at 3 days and CD8^+^ cells at 7 days post-infection were higher than those of other groups, respectively. These results show that CD8^+^ cells may act anti-viral function during influenza infection. We could not recognized whether these were Th1 or Th2 lymphocytes, however both CD4^+^ and CD8^+^ cells may play important roles in pathophysiology of A(H1N1)pdm09-infected asthmatic patients.

The histopathological findings in the early phase of infection in asthmatic/A(H1N1)pdm09 mice were severe pneumonia with abscess formation, and were not observed in any other groups (Fig. 4 **)**.

These results demonstrated that pulmonary inflammation in asthmatic mice is induced beginning in the early phase of A(H1N1)pdm09 infection, which mirrors the finding that A(H1N1)pdm09 infection in asthmatic children induces severe pulmonary complications, including pneumonia, atelectasis, etc., after a shorter incubation period than with seasonal virus infection. After A(H1N1)pdm09 infection, the viral loads in BAL fluid from asthmatic mice were higher than those from control mice, which was not typically observed after seasonal infection (Fig. 3). Immunostaining of the virus showed many InfA-NP-positive cells in the lungs of asthmatic/A(H1N1)pdm09 and control/A(H1N1)pdm09 mice since early phase after viral infection, but not in the lungs of mice from seasonal H1N1 groups, as shown in Fig. 5. Previous reports demonstrated that A(H1N1)pdm09 viral proteins were detected in damaged type II pneumocytes, epithelial cells, and infiltrated macrophages in the lung by immunohistochemistry^16–18^. In addition, at autopsy after A(H1N1)pdm09 infection, acute diffuse alveolar damage was observed^19^. Avian influenza viruses preferentially bind to SAα2-3 Gal, which is expressed on distal bronchioles and type II pneumocytes in the lower respiratory tract^19^. In contrast, seasonal H1N1 influenza viruses bind to SAα2-6 Gal, which is expressed on epithelial cells in the upper respiratory tract. A(H1N1)pdm09 virus binds to both SAα2-6 Gal and SAα2-3Gal^19, 20^. Predominant replication of A(H1N1)pdm09 virus in the lower respiratory tract, compared with that of seasonal influenza virus, could explain the distinct viral loads shown in Fig. 3. These findings suggested that A(H1N1)pdm09 virus may induce severe pneumonia in asthmatic patients, which is much less likely in seasonal influenza-infected asthmatic patients or non-asthmatic patients. We concluded that severe pulmonary complications are caused not only by the characteristics of the infecting viruses but also by factors in the host defence of asthmatic children during A(H1N1)pdm09 infection.

In the present study, cytokine levels in BAL fluid (Fig. 1) appeared not to be associated with the lung histopathology. The histopathological analyses showed that airway inflammation was augmented in asthmatic mice when compared to control mice infected with either A(H1N1)pdm09 or seasonal influenza (Fig. 4). However, BAL fluid cytokine levels showed no paralleled alterations. In fact, inflammatory cytokine levels in the non-infected groups were equivocally low in mice with or without bronchial asthma, which suggested that these histopathological changes without detectable cytokine elevations, were independent of asthma.

At 3 days post-infection, IFN-γ levels in BAL fluid were significantly higher in the asthmatic/A(H1N1)pdm09 group than in the control/A(H1N1)pdm09 group, in contrast to the pattern at 7 days post-infection. A previous report also showed that IFN-γ levels in asthmatic mice were lower than those in control mice at 7 days after A(H1N1)pdm09 infection^21^. Virus titres in the asthmatic/A(H1N1)pdm09 group at both 3 and 7 days post-infection were significantly higher than those in control mice, and the titres of asthmatic/A(H1N1)pdm09 mice at 3 days post-infection were the highest among all groups at both 3 and 7 days post-infection. In contrast, the virus titres of the control/seasonal group were significantly lower than those of the asthmatic/seasonal group at both 3 and 7 days post-infection. IFN-γ levels were reportedly reduced in bronchial asthmatic patients, indicating an alteration in the cytokine milieu, with excess production of Th2 cytokines and decreased production of Th1 cytokines^22, 23^, and it has been reported that bronchial asthma patients show suppressed innate immunity^24–26^. IFN-γ is produced by Th1 cells, CD8^+^T (cytotoxic T) cells, natural killer (NK) cells, and NKT cells^27, 28^, and in our study, IFN-γ levels were elevated against the high virus load in the asthmatic/A(H1N1)pdm09 group at 3 days post-infection, but were not sufficiently augmented at 7 days post-infection. Pulmonary inflammation, through not only the IFN-γ pathway but also other inflammatory molecules, might be involved in the exacerbation observed in A(H1N1)pdm09-infected asthma patients.

We have some limited reasons for the unexplainable reciprocal pattern of virus titres in A(H1N1)pdm09- and seasonal influenza-infected asthmatic mice. The viral titre may depend on both the specificity of these viruses and the distinctive host defences in asthmatic individuals. However, further investigations are needed to characterize the immune responses against A(H1N1)pdm09 infection in asthmatic patients.

In this study, the lung tissues of asthmatic/A(H1N1)pdm09 and control/A(H1N1)pdm09 mice were positive for InfA-NP antigens, whereas the lung tissues of mice in seasonal groups were not, even though virus titres were detected in the BAL fluid of all infected groups. The reasons for this discrepancy may be the lower affinity for the virus receptors present in the lower respiratory tract or some yet unknown properties of the polyclonal antibodies and/or viral strains used, although the reason remains unclear. Further research should be directed toward immunohistochemical studies of the upper respiratory tract along with lung function and airway hyperresponsiveness.

In conclusion, A(H1N1)pdm09 infection can induce more severe pulmonary inflammation in patients with bronchial asthma than seasonal H1N1 infection, based on the dynamics of early excessive production of inflammatory cytokines and the reciprocal depression of anti-viral cytokines, along with high viral loads in a mouse model of bronchial asthma.

## Methods

### Sensitization of mice and allergen challenge

BALB/c mice (age: 6–8 weeks) were obtained from Chiyoda Kaihatsu Co., Ltd. (Tokyo, Japan) and were sensitized and challenged with grade II ovalbumin (OVA; Sigma-Aldrich., St. Louis, MO, USA), as previously described^14, 15^. All animal procedures were approved by the Institutional Animal Care and Use Committee of Yamaguchi University (No. 29-S01), and all methods were conducted in accordance with the approved guidelines. This study was performed independently of our previous reports^14, 15^.

### Viruses, infection of mice, and preparation of BAL fluid

Mouse-adapted A(H1N1)pdm09 (strain: A/Narita/1/09) or seasonal H1N1 (strain: A/Puerto Rico) viruses were provided by the National Institute of Infectious Diseases (Tokyo, Japan). On day 31, influenza virus (concentration: 1 × 10^5^ pfu/20 μL) or vehicle (mock-infection) was administered intranasally to mice. Then, mice were euthanized at 2, 3, or 7 days post-infection, and samples were collected.

BAL fluids were collected on day 33, 34, and 38 (2, 3, and 7 days post-infection) with three consecutive 1-mL instillations of phosphate-buffered saline (PBS) at room temperature. The collected BAL fluid was centrifuged at 1,500 rpm for 5 min at 4 °C, and the supernatants were stored at −80 °C for estimation of cytokine levels and virus titres.

### Cytokine assays

The concentrations of various cytokines, including IL-1β, IL-2, IL-4, IL-6, IL-10, IL-17A, IFN-γ, and TNF-α, in BAL fluid were measured using the Cytometric Bead Array (CBA) Kit (BD Biosciences, San Diego, CA, USA). The levels of IL-1β and IL-13 in BAL fluid were measured using an ELISA kit (R&D systems, Minneapolis, MN, USA) according to the manufacturer’s instructions. The lower detection limits for IL-1β, IL-2, IL-4, IL-6, IL-10, IL-17A, IFN-γ, TNF-α, and IL-13 were 2.31, 0.1, 0.03, 1.4, 16.8, 0.8, 0.5, 0.9, and 1.5 pg/mL, respectively.

### Measurement of CD4^+^ cells, CD8^+^ cells, eosinophils, and neutrophils in BAL fluid

Cell pellets were resuspended in PBS and stained with fluorescein isothiocyanate (FITC)-conjugated anti-CD4 (BD Biosciences) and allophycocyanin (APC)-conjugated anti-CD8 (BD Biosciences) antibodies; erythrocytes were lysed by the addition of FACS Lysing Solution (Becton Dickinson, San Diego, CA, USA). The cell suspensions were centrifuged, and the cell pellets were resuspended in PBS containing sodium azide and paraformaldehyde. Then, the cells were analysed with a FACSCalibur flow cytometer (Becton Dickinson) equipped with CellQuest software (Becton Dickinson). Cytospin samples were prepared using Auto Smear CF-12D (Sakura Co., Tokyo, Japan), and cellular infiltration in BAL fluid was assessed on Wright-Giemsa-stained slides (Wako Pure Chemical Industries, Ltd.).

### Plaque assay

Plaque assays were performed as described previously^10, 11^. Briefly, Madin-Darby canine kidney (MDCK) cells (Lonza, Walkersville, MD, USA) were maintained at 37 °C in a humidified 5% CO_2_ chamber under stationary conditions. Each well of a 6-well plate was seeded with 1 × 10^6^ cells and cultured in α-minimum essential medium (MEM; GIBCO/Invitrogen, Carlsbad, CA, USA) containing 10% foetal bovine serum (FBS), 100 units/mL penicillin (GIBCO), and 100 μg/mL streptomycin (GIBCO). After two washes with serum-free Dulbecco’s modified Eagle’s medium (DMEM; GIBCO/Invitrogen), the cells were maintained in serum-free DMEM at 37 °C for 1 h. Then, each well was overlaid with 200 μL of diluted BAL (10^−3^, 10^−4^, and 10^−5^ dilutions) and incubated at 37 °C for 1 h. After one wash in serum-free DMEM, the cells were overlaid with serum-free DMEM containing 0.8% agarose (Becton, Dickinson and Company, Sparks, MD, USA), 0.1% diethylaminoethyl-Dextran (Sigma-Aldrich), and 7 μg/mL trypsin (Sigma-Aldrich). The cells were cultured at 37 °C for 72 h, fixed in 10% formaldehyde (Wako Pure Chemical Industries, Ltd., Osaka, Japan), and then stained with 0.037% methylene blue (Wako Pure Chemical Industries, Ltd.). Each experiment was performed in duplicate.

### Histological and immunohistochemical examination of the lungs

Lung tissues were fixed in 10% buffered formalin for 24 h at room temperature and then embedded in paraffin. Serial sections (3-µm thick) were cut and stained with hematoxylin and eosin (H&E; Muto Pure Chemicals Co., Ltd., Tokyo, Japan). The distribution of viral antigens was examined by immunological staining with a rabbit polyclonal antibody against InfA-NP, which recognize not only A(H1N1)pdm09 and seasonal H1N1, but also H3N2 influenza^29^. Specific antigen-antibody reactions were visualized by 3,3′-diaminobenzidine tetrahydrochloride staining with the Envision Rabbit/HRP system (DAKO Cytomation). The stained sections were observed by light microscopy to evaluate the degree of pulmonary inflammation and localization of A(H1N1)pdm09-infected cells.

### Statistical analysis

The differences between groups were analysed by the Mann-Whitney *U* test. P values less than 0.05 were considered statistically significant. All analyses and calculations were performed using SPSS version 11.0 (SPSS Inc., Chicago, IL, USA).
